# A cortical gradient in area 55b links language and somato-cognitive action networks

**DOI:** 10.21203/rs.3.rs-9934100/v2

**Published:** 2026-08-17

**Authors:** Guowei Wu, Xiuyi Wang, Baihan Lv, Yi Du

**Affiliations:** 1 State Key Laboratory of Cognitive Science and Mental Health, Institute of Psychology, Chinese Academy of Sciences, Beijing, China.; 2 Department of Psychology, University of Chinese Academy of Sciences, Beijing, China.

**Keywords:** area 55b, language, sensorimotor–semantic interface

## Abstract

How the cortex converts high-level cognition into action remains unresolved. We identify left frontal area 55b as a graded transition between the canonical language network and the somato-cognitive action network (SCAN). Across individualized fMRI mapping of word listening, reading, speaking and writing, resting-state connectivity, diffusion tractography and independent datasets, 55b contained a reproducible anterior–posterior gradient. Its anterior end aligned and structurally connected with the language network and carried stronger semantic-category information, whereas its posterior end aligned and connected with SCAN and preferentially encoded production and articulatory features. Production further reweighted model-estimated 55b coupling toward frontal language and speech- and hand-motor regions. Area 55b also occupied convergent transitions across multiple cortical hierarchies. These findings show how a macroscale relationship between cognitive and action networks can be implemented within a small cortical territory, providing a general mechanism for flexible cognition-to-action transformation.

## Introduction

The cerebral cortex must solve a general integration problem: representations distributed across association cortex have to guide actions implemented by modality- and effector-specific systems. Network neuroscience has mapped many of these operations onto large-scale systems, but maps alone do not explain how information crosses network boundaries. Cortex is not organized solely as a mosaic of discrete modules. Its microstructure, connectivity, metabolism, and function vary along graded axes extending from primary sensorimotor to transmodal association cortex^1 4^. A key unresolved question is therefore how macroscale gradients are translated into local organization where cognitive and action systems meet. One possibility is that network interfaces are themselves graded: neighboring parts of a small cortical territory may differ systematically in network alignment and representational content, allowing integration without erasing functional specialization.

Language provides a demanding model in which to test this possibility. A single concept can be reached from auditory speech or visual text and can guide spoken or written action. These operations require coordination between systems that represent linguistic and conceptual content ^5–12^ and systems that encode perceptual forms, action plans, and effector-specific motor commands ^13–18^. The recently described somato-cognitive action network (SCAN) adds an important dimension to this architecture. SCAN regions interleave with classical effector representations in motor cortex and have been proposed to integrate goals, bodily state, and coordinated actions rather than control a single effector^19–21^. The canonical language network and SCAN approach one another in lateral frontal cortex, creating a candidate junction at which linguistic representations could be reformatted for action. Whether this junction is a hard network boundary, a nonspecific overlap, or an organized cortical gradient is unknown.

Area 55b is anatomically and functionally well positioned to address this question. Defined in a multimodal cortical atlas as a small region of dorsal lateral frontal cortex ^22^, 55b varies substantially across individuals and is easily blurred by group averaging ^23–27^. Its functional identity has also remained unsettled. Studies of speech production, lesions, and neurosurgical mapping implicate this territory in articulatory planning and motor sequencing ^17,28–31^, whereas connectivity and task studies also associate it with the language network and semantic processing ^32–36^. These accounts are often treated as competing assignments of an indivisible region to either language or motor function. An alternative is that the apparent inconsistency reflects genuine internal organization: one end of 55b may be more strongly embedded in the language network, whereas the other is more strongly embedded in SCAN. Testing this account requires subject-sensitive mapping and convergent measurements of network alignment, representational content, anatomical connectivity, and task-dependent communication.

We therefore asked whether left area 55b contains a reproducible internal gradient that links the language network with SCAN, and whether this gradient has consequences for the transformation between linguistic content and action. Participants performed four word-level tasks spanning two input modalities (listening and reading) and two output effectors (speaking and writing). We combined individualized functional parcellation with multivariate decoding and encoding, resting-state connectivity gradients, diffusion tractography, and dynamic causal modelling. Independent Peking University^37^ and Human Connectome Project datasets ^38,39^ were used to test the reproducibility of lexical recruitment and local gradient organization. We predicted that anterior 55b would show stronger language-network alignment, semantic-category information, and structural connectivity with the language network, whereas posterior 55b would show stronger SCAN alignment, production bias, articulatory-feature encoding, and structural connectivity with SCAN. We further asked whether production reweights model-estimated 55b coupling toward frontal and effector-specific motor systems and whether 55b occupies a transition across established cortical hierarchies.

The results reveal a coherent anterior–posterior organization. Anterior 55b preferentially aligned with the language network and carried stronger semantic-category information, whereas posterior 55b preferentially aligned with SCAN and carried stronger production-related and articulatory information. A crossed pattern of white-matter connectivity mirrored this functional differentiation, and production shifted model-estimated directed coupling toward speech and hand motor cortices. At the macroscale, 55b lay within a convergent transition from sensorimotor-adjacent to association-adjacent cortex. Thus, rather than assigning 55b exclusively to either language or action, our findings identify it as a graded cross-network transition. More broadly, they show how a macroscale relationship between cognitive and action networks can be implemented within a small cortical territory.

## Results

### A multimodal lexical network identifies area 55b at the junction of language and action systems

**Fig. 1 |:**
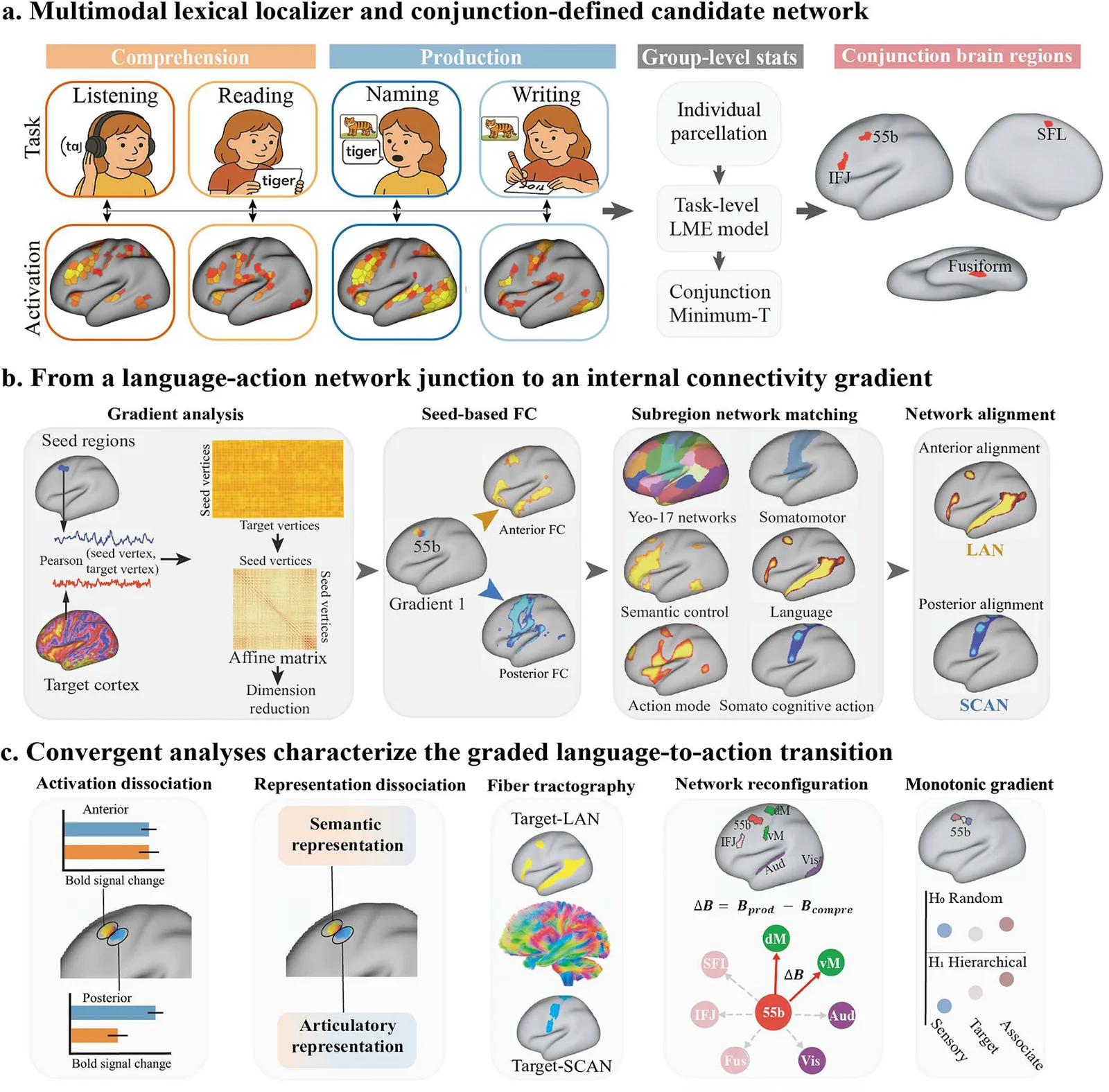
Multimodal localization and analysis framework identify area 55b as a candidate language–action transition. **a**, Multimodal lexical localizer and conjunction-defined candidate network. Thirty participants completed word listening, reading, picture naming and picture writing in three sessions, each with a modality-matched control. Linear mixed-effects models were fitted in individualized 900-parcel maps and corrected across parcels using FDR at *q* < 0.05. A minimum-T conjunction across the four word > control maps identified area 55b, IFJ, SFL and anterior fusiform gyrus. **b**, Area 55b lies where the canonical language network approaches the Somato-cognitive action network (SCAN). Whole-cortex connectivity profiles of 55b vertices were converted to a cosine-similarity affinity matrix; diffusion embedding yielded the dominant gradient, and subject-level gradients were aligned to the group solution. The median split was used only for operational comparison of the gradient ends. **c**, Gradient-defined sectors were characterized using task responses and cross-modal classification, semantic-category decoding and articulatory-feature encoding, tractography, 55b-centred DCM with parametric empirical Bayes (PEB), and six macroscale cortical hierarchy maps. The bottom band summarizes the hypothesized continuous transition; empirical tests are presented in Figs. 2–5. Panels b and c are workflow schematics.

A conjunction analysis across word listening, reading, naming, and writing—each contrasted with a modality-matched control—identified four regions with significantly greater lexical responses: area 55b, the superior frontal language area (SFL), the inferior frontal junction (IFJ), and the anterior fusiform gyrus (FDR-*q* < 0.05; Fig. 1a and Table 1). The four task–control contrasts, individualized parcel-wise models, and minimum-T conjunction are summarized in Fig. 1a. Recruitment across two sensory inputs and two motor outputs established a modality-invariant candidate network without presupposing a unique interface region.

The same network was recovered in a vertex-wise conjunction analysis (**Supplementary Fig. 3**). In the independent Peking University dataset, area 55b, IFJ, and anterior fusiform gyrus were recruited during both comprehension and production, whereas SFL showed significant comprehension activation but weaker production activation (**Supplementary Results** and **Supplementary Fig. 4**). Among these regions, area 55b occupied the anatomical junction between the language network and SCAN (Fig. 1b). We therefore tested whether this apparent overlap concealed an organized internal gradient rather than a functionally uniform zone. Figure 1b and 1c summarize the connectivity-gradient construction and the convergent functional, representational, structural, effective-connectivity^40^, and cortical-hierarchy analyses ^41^ used to characterize that gradient.

### A reproducible internal gradient differentiates language-aligned and SCAN-aligned sectors of 55b

We derived the dominant local connectivity gradient within the Glasser-defined 55b territory and used a median split to compare its ends. The split provided an operational anterior–posterior comparison; the underlying organization was interpreted as continuous rather than as two new cortical modules.

**Fig. 2 |:**
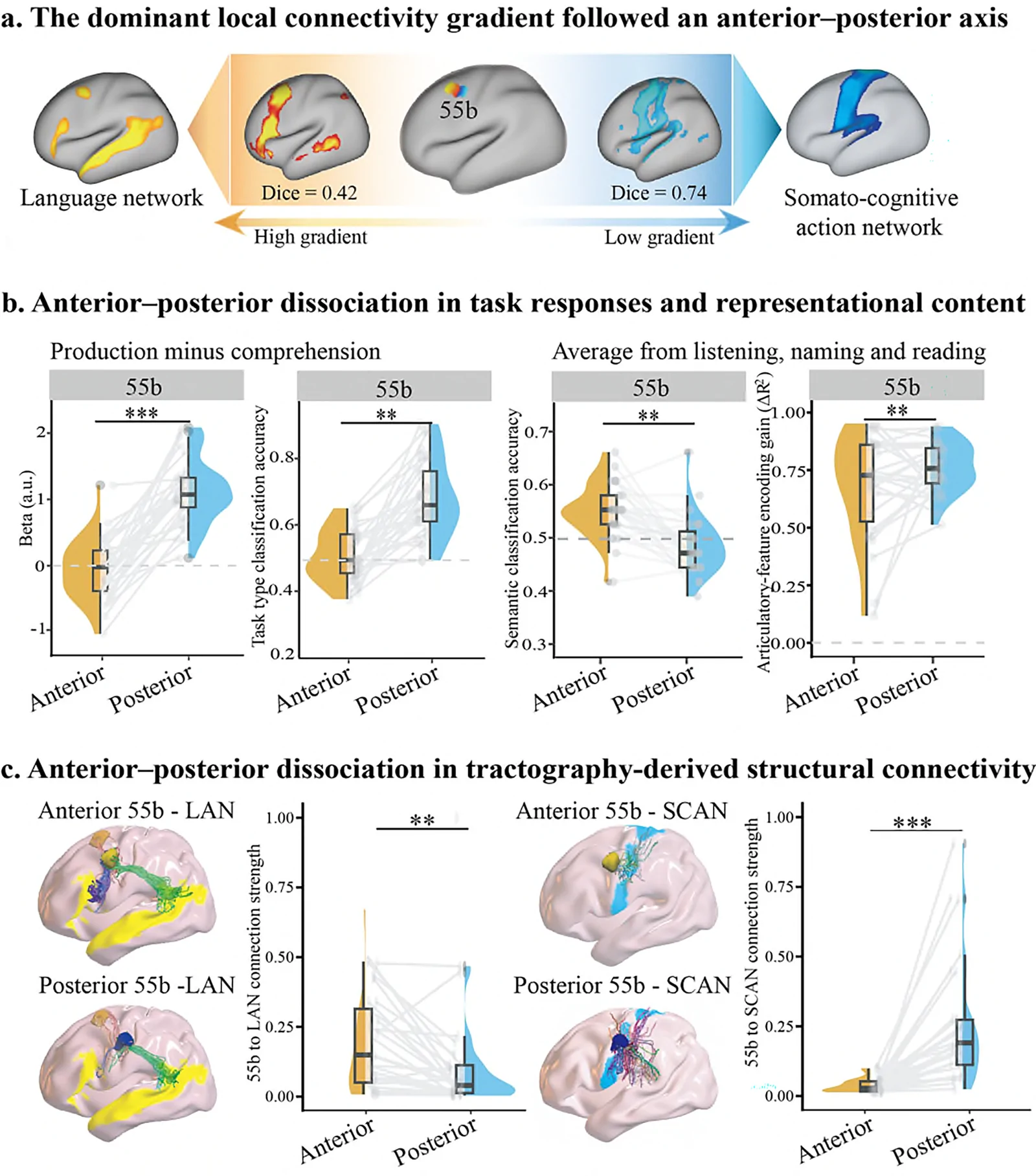
A reproducible internal gradient in area 55b links network alignment, functional specialization, and crossed white-matter connectivity. **a**, The dominant local connectivity gradient followed an anterior–posterior axis. Anterior high-gradient 55b aligned most strongly with the canonical language network (Dice = 0.42), whereas posterior low-gradient 55b aligned most strongly with SCAN (Dice = 0.74); independent replication is shown in Supplementary Fig. 5. **b**, Functional differentiation. Posterior 55b showed stronger production bias, comprehension–production classification, and articulatory-feature encoding, whereas anterior 55b showed stronger semantic-category decoding. **c**, Tractography-derived connectivity. Anterior 55b showed stronger normalized streamline-count connectivity with the language network (LAN), whereas posterior 55b showed stronger connectivity with SCAN. Box–violin plots show medians and interquartile ranges; violin widths indicate density; grey lines connect paired participant estimates. Dashed lines indicate the relevant null value. Asterisks denote FDR-corrected significance (*q < 0.05, **q < 0.01, ***q < 0.001).

The dominant gradient followed an anterior–posterior axis (Fig. 2a). Among the tested network templates, anterior high-gradient 55b aligned most strongly with the canonical language network (Dice = 0.42), whereas posterior low-gradient 55b aligned most strongly with the somato-cognitive action network (SCAN; Dice = 0.74). The same gradient orientation and preferential network alignment were reproduced in the Human Connectome Project and Peking University datasets (**Supplementary Fig. 5**), establishing a stable local transition from language-aligned to action-aligned connectivity.

### Functional specialization along the 55b gradient is mirrored by crossed white-matter connectivity

The connectivity gradient was accompanied by systematic functional differentiation. As shown in Fig. 2b (left two panels), posterior 55b showed a larger production–comprehension response difference than anterior 55b (*t*(29) = 10.01, FDR-*q* < 0.001, Cohen’s *d* = 1.83) and stronger multivariate discrimination of the two task domains (*t*(29) = −3.64, FDR-*q* = 0.005, Cohen’s *d* = −0.67). Considered separately, anterior 55b showed neither a production bias (mean *β* = 0.01; *t*(29) = 0.20, FDR-*q* = 0.84) nor above-chance task-domain classification (mean = 0.52, *SD* = 0.09; *t*(29) = 0.92, FDR-*q* = 0.46), whereas posterior 55b showed both a strong production bias (mean *β* = 0.94; *t*(29) = 10.24, FDR-*q* < 0.001) and above-chance classification (mean = 0.72, *SD* = 0.13; *t*(29) = 8.93, FDR-*q* < 0.001).

Representational measures showed a complementary dissociation. Semantic-category decoding was greater in anterior than posterior 55b (anterior mean = 0.53, *SD* = 0.11; posterior mean = 0.48, *SD* = 0.10; *t*(29) = 2.74, FDR-*q* = 0.007), whereas articulatory-feature encoding was greater posteriorly (posterior mean = 0.76, *SD* = 0.11; anterior mean = 0.59, *SD* = 0.37; *t*(29) = −2.60, FDR-*q* = 0.007; Fig. 2b right two panels). Because the articulatory model was compared with a word-onset baseline, this measure is interpreted as articulatory-feature-related encoding rather than a uniquely phonetic code.

Tractography-derived connectivity mirrored this functional gradient (Fig. 2c). A 2 × 2 repeated-measures ANOVA revealed a network × subregion interaction (*F*(1,25) = 30.59, *P* < 0.001, generalized *η*^*2*^ = 0.223). Anterior 55b showed stronger normalized streamline-count connectivity with the language network than posterior 55b (anterior – posterior = 0.013; *t*(25) = 3.35, *P* = 0.003, Cohen’s *d* = 0.66), whereas posterior 55b showed stronger connectivity with SCAN than anterior 55b (anterior – posterior = −0.283; *t*(25) = −5.35, *P* < 0.001, Cohen’s *d* = −1.05). Thus, network alignment, representational content, task preference, and estimated white-matter connectivity converged on the same anterior language-facing to posterior action-facing organization.

### Area 55b distinguishes comprehension from production across sensory and motor formats

**Fig. 3 |:**
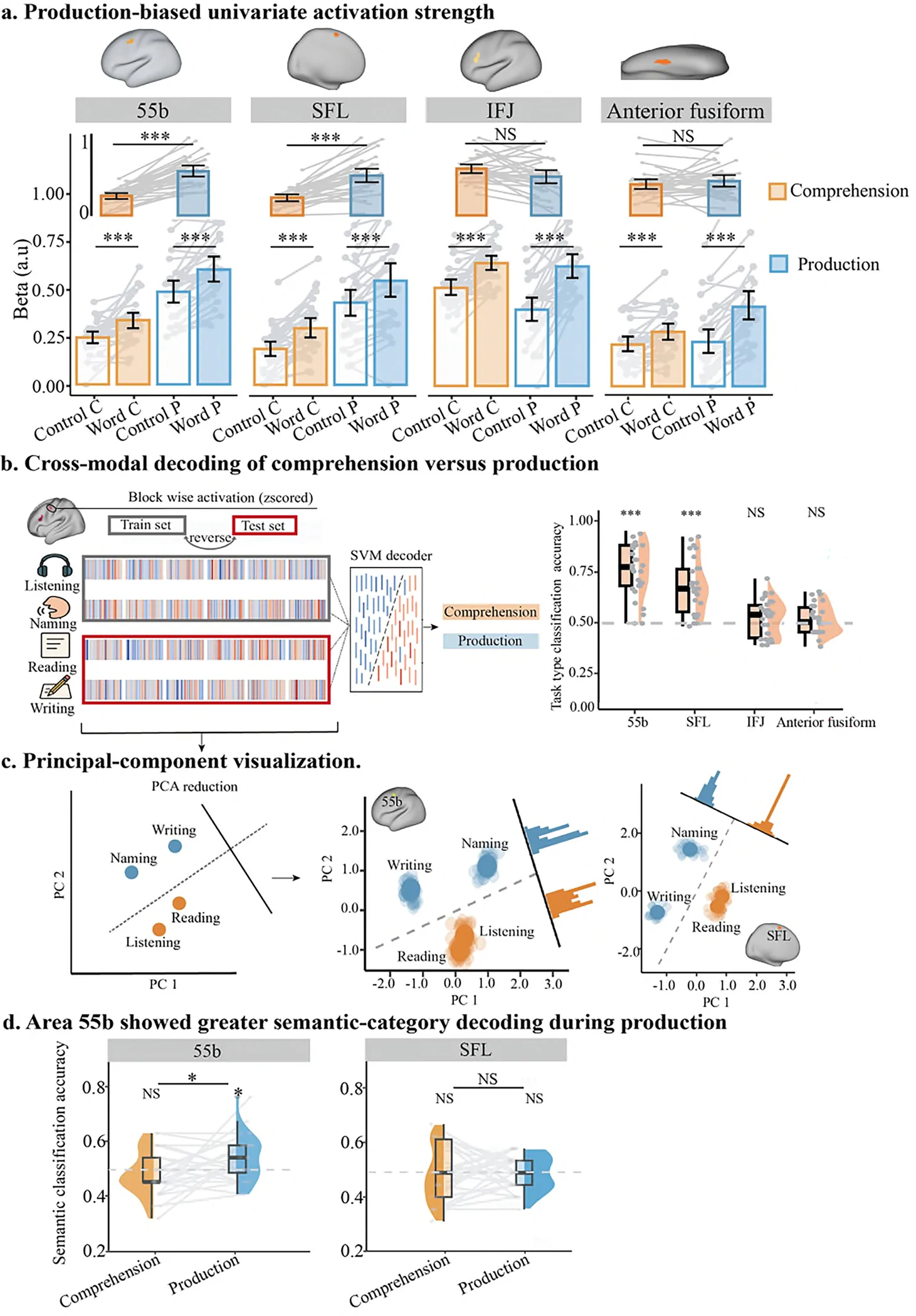
Area 55b differentiates word comprehension from production across sensory and motor formats. **a**, Production-biased univariate responses. Area 55b and SFL showed stronger responses during production than comprehension. **b**, Cross-modal classification. Area 55b and SFL showed above-chance classification of comprehension versus production, whereas IFJ and anterior fusiform gyrus did not. **c**, Principal-component visualization. Comprehension and production occupied separable positions in area 55b and SFL. **d**, Area 55b showed greater semantic-category decoding during production than comprehension, with above-chance decoding only during production; SFL showed neither effect. Box–violin plots show participant-level values, medians, and interquartile ranges; error bars denote SEM; dashed lines indicate chance or zero. Asterisks indicate corrected significance (***P < 0.001, **P < 0.01, *P < 0.05); NS, not significant.

We next tested whether the gradient-defined territory distinguished the direction of language processing across modalities. In univariate responses, area 55b and SFL showed production-biased profiles (area 55b: *t*(29) = 9.226, *P* < 0.001, Cohen’s *d* = 1.68; SFL: *t*(28) = 6.752, *P* < 0.001, Cohen’s *d* = 1.25; Fig. 3a). IFJ and anterior fusiform gyrus showed no significant production–comprehension difference (IFJ: *t*(29) = −2.272, *P* = 0.153, Cohen’s *d* = −0.415; anterior fusiform gyrus: *t*(28) = 2.729, *P* = 0.054, Cohen’s *d* = 0.51). *P* values were Bonferroni-corrected. Production-biased responses in area 55b and SFL were replicated in the Peking University dataset (**Supplementary Fig. 4**).

Cross-modal classifiers were trained on one comprehension–production pair and tested on another pair involving different sensory and motor formats. Area 55b and SFL showed above-chance classification (area 55b: mean accuracy = 0.76, *SD* = 0.13; *t*(29) = 10.74, *P* < 0.001, Cohen’s *d* = 1.96; SFL: mean accuracy = 0.67, *SD* = 0.13; *t*(29) = 7.494, *P* < 0.001, Cohen’s *d* = 1.37), with higher accuracy in area 55b than SFL (*t*(29) = 3.05, *P* = 0.005, Cohen’s *d* = 0.56; Fig. 3b). IFJ and anterior fusiform gyrus did not show above-chance classification.

Principal-component visualization showed the same task-domain separation in area 55b and SFL (Fig. 3c). An orthographic control did not distinguish written-form tasks from non-orthographic tasks in either region (area 55b: *P* = 1.00, Cohen’s *d* = −0.12; SFL: *P* = 1.00, Cohen’s *d* = −0.09; Supplementary Results). This control argues against simple grouping by orthographic form, but does not eliminate the picture-versus-word difference between production and comprehension. The result is therefore interpreted as a cross-modal task-domain distinction rather than a fully format-invariant computation.

Area 55b, but not SFL, also carried stronger semantic-category information during production (Fig. 3d): production decoding exceeded comprehension decoding (*Δaccuracy* = 0.06; *t*(28) = 2.74, FDR-*q* = 0.045, Cohen’s *d* = 0.51) and chance (mean = 0.54, *SD* = 0.08; FDR-*q* = 0.023, Cohen’s *d* = 0.48), whereas comprehension decoding did not exceed chance (mean = 0.49, *SD* = 0.08; FDR-*q* = 0.82, Cohen’s *d* = −0.18). SFL showed neither effect. Because production was picture-cued, the result indexes available category information rather than necessarily establishing an amodal semantic code.

### Production reweights 55b coupling toward frontal language and motor-effector systems

**Fig. 4 |:**
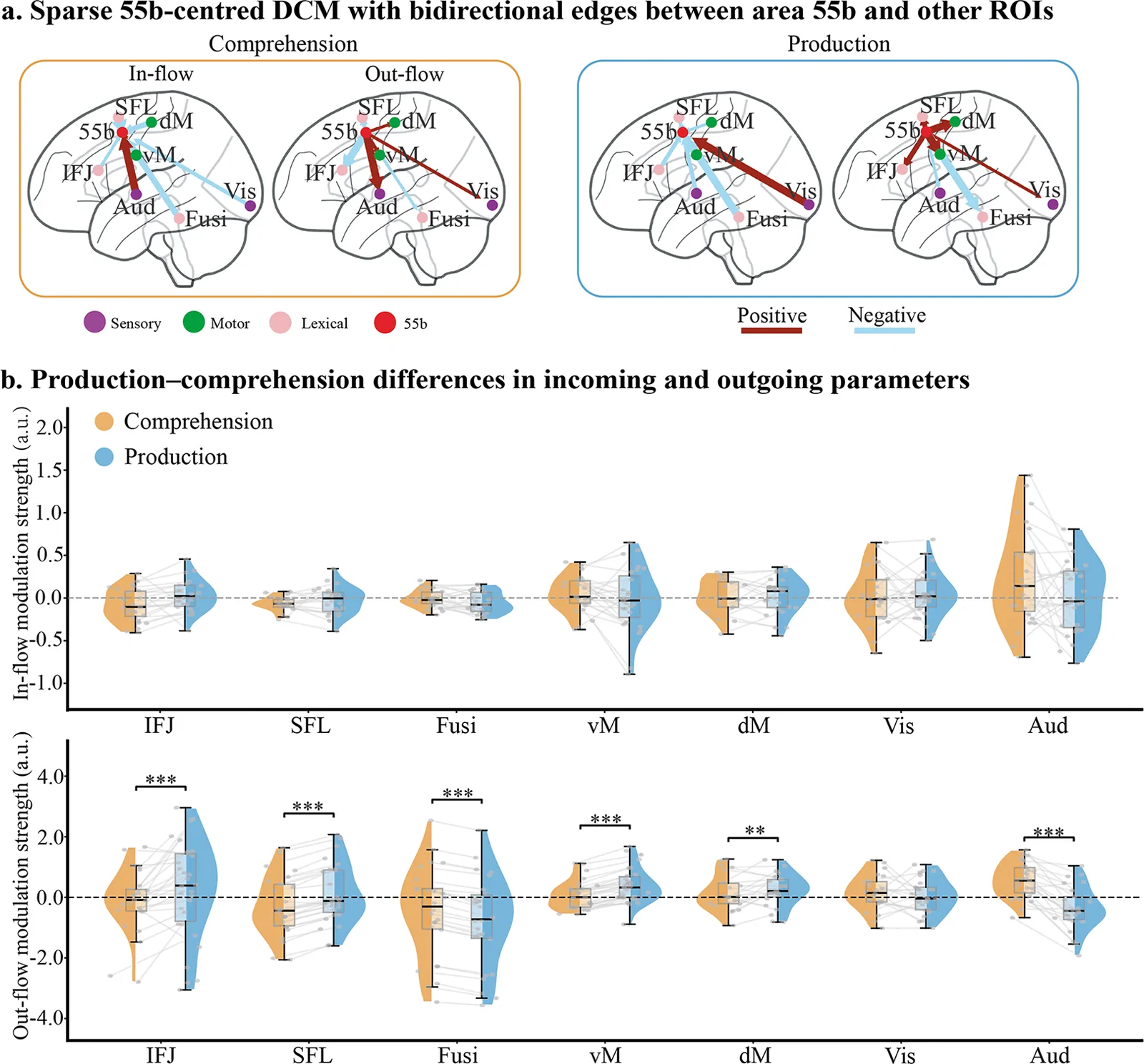
Task-dependent model-estimated directed coupling of area 55b. **a**, Sparse 55b-centred DCM with bidirectional edges between area 55b and sensory, lexical/language-related, and effector-specific motor regions. Arrows show model-estimated directed coupling; line thickness scales with the absolute group-mean modulation parameter; red and light blue denote positive and negative modulation. **b**, Production–comprehension differences in incoming and outgoing parameters. Incoming edges showed no significant task-domain differences after FDR correction, whereas outgoing edges were reweighted during production toward frontal language-related and motor regions. Asterisks denote FDR-corrected significance (*q < 0.05, **q < 0.01, ***q < 0.001); NS, not significant. Abbreviations: Aud, auditory cortex; Vis, visual cortex; Fusi, anterior fusiform gyrus; SFL, superior frontal language area; IFJ, inferior frontal junction; vM, ventral laryngeal–orofacial motor cortex; dM, dorsal hand motor cortex.

We then tested whether language production and comprehension were associated with different model-estimated directed coupling between area 55b and task-relevant sensory, lexical, and motor regions. A sparse, 55b-centred dynamic causal model estimated bidirectional edges between 55b and auditory cortex, visual cortex, SFL, IFJ, anterior fusiform gyrus, ventral laryngeal–orofacial motor cortex, and dorsal hand motor cortex, without modelling direct connections among the non-55b nodes (Fig. 4a). The architecture was hypothesis-constrained and was not designed to establish a unique whole-network causal pathway.

Relative to comprehension, production increased outgoing model-estimated coupling from 55b to ventral laryngeal–orofacial motor cortex (mean difference = 0.324; *t*(29) = 11.40, FDR-*q* < 0.001, Cohen’s *d* = 2.08), dorsal hand motor cortex (mean difference = 0.302; *t*(25) = 3.63, FDR-*q* = 0.002, Cohen’s *d* = 0.71), IFJ (mean difference = 0.746; *t*(29) = 5.00, FDR-*q* < 0.001, Cohen’s *d* = 0.91), and SFL (mean difference = 0.413; *t*(29) = 15.97, FDR-*q* < 0.001, Cohen’s *d* = 2.92; Fig. 4b).

The reweighting was selective: 55b-to-visual cortex did not differ between task domains, whereas 55b-to-auditory cortex and 55b-to-fusiform coupling were stronger during comprehension. None of the seven incoming edges to 55b differed reliably after FDR correction (Fig. 4b and **Supplementary Table 1**). Within the specified model, production therefore preferentially weighted outgoing 55b-centred coupling toward frontal language-related and motor-effector systems.

### Area 55b occupies a convergent transition across macroscale cortical hierarchies

**Fig. 5 |:**
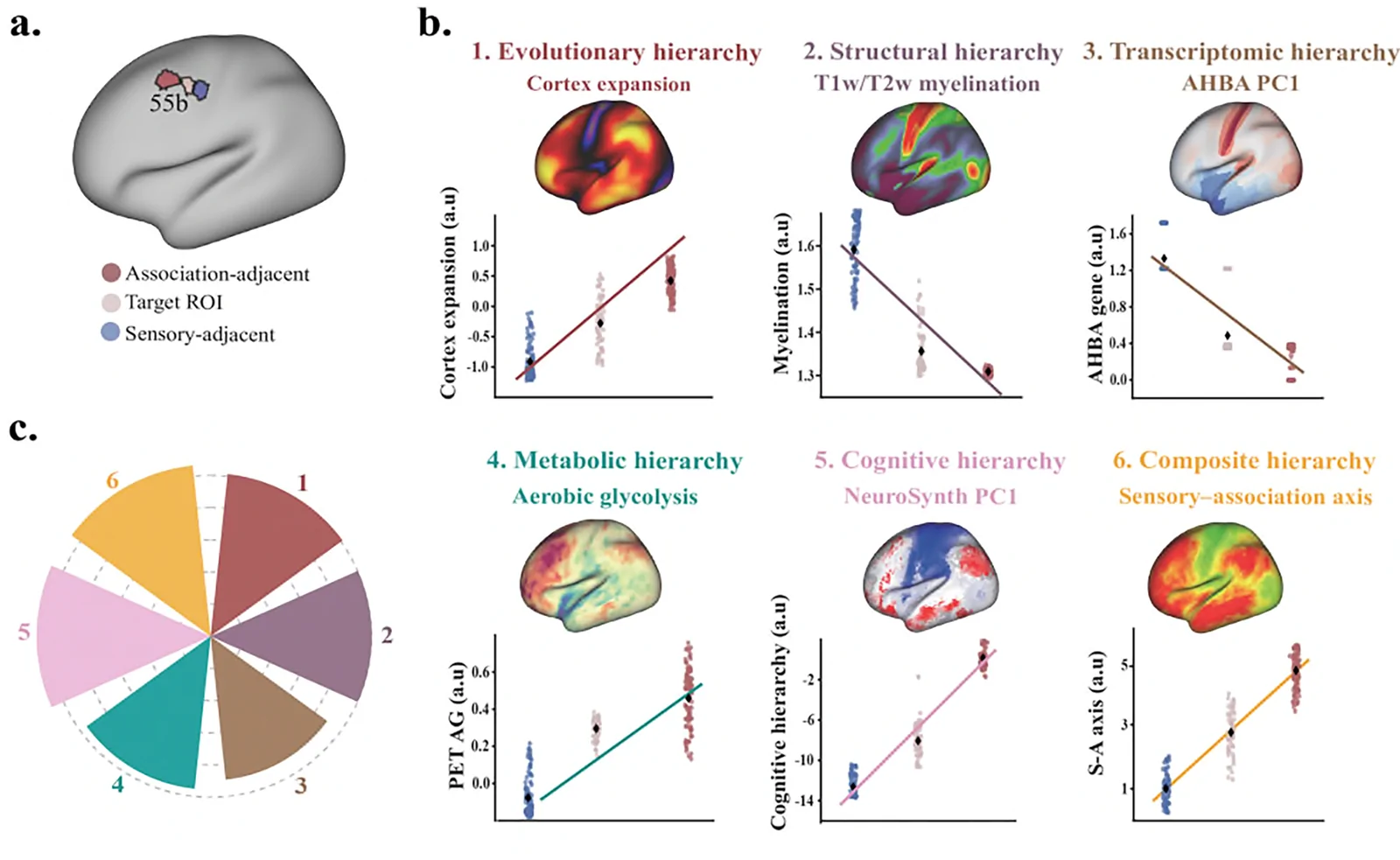
Area 55b occupies a convergent transition across macroscale cortical hierarchies. **a,** Three-parcel sampling of sensorimotor-adjacent cortex (blue), the parcel containing area 55b (beige), and association-adjacent cortex (red). **b**, Ordered parcel values across evolutionary expansion, T1w/T2w myeloarchitecture, and AHBA gene-expression PC1. **c**, Multidimensional transition profile and ordered parcel values for PET-derived aerobic glycolysis, NeuroSynth PC1, and a composite sensory-to-association hierarchy. The analysis establishes regional embedding around 55b and should not be interpreted as direct vertex-wise correspondence between every hierarchy map and the internal functional gradient.

We finally placed the 55b territory within broader principles of cortical organization. For each of six independent hierarchy maps, we sampled vertex-wise values from three adjacent parcels spanning sensorimotor-adjacent cortex, the parcel containing area 55b, and association-adjacent cortex (Fig. 5a). The maps captured evolutionary, myeloarchitectonic, transcriptomic, metabolic, meta-analytic functional, and composite sensory-to-association dimensions (Fig. 5b,c; Methods). This analysis establishes the regional position of 55b within a local transition; it does not test direct vertex-wise alignment between each hierarchy map and the internal 55b connectivity gradient.

Across all six maps, values progressed monotonically from the sensorimotor-adjacent parcel through the 55b parcel to the association-adjacent parcel (all ordered-trend tests *P* < 0.0001; Fig. 5b,c). The multidimensional transition profile around 55b was stronger and more consistent than those around the other conjunction-defined regions and canonical language-network controls (**Supplementary Fig. 7** and **Supplementary Table 2**). The hierarchy analysis therefore embeds the local 55b finding within a broader sensory-to-association landscape while keeping the regional and within-territory levels of evidence distinct.

## Discussion

Large-scale brain networks must remain functionally distinct while exchanging information rapidly enough to turn cognition into action. Our findings reveal a local cortical organization that can support both requirements. Within left area 55b, a reproducible anterior–posterior connectivity gradient separated a language-aligned end from an action-aligned end. Anterior 55b showed stronger semantic-category information and preferential tractography-derived connectivity with the language network, whereas posterior 55b showed stronger production bias, articulatory-feature encoding, and preferential connectivity with SCAN. During production, 55b also reweighted model-estimated directed coupling toward frontal language regions and laryngeal–orofacial and hand motor cortices. The territory occupied a convergent local transition across multiple macroscale cortical hierarchies. Together, these observations suggest that 55b is neither a uniform language module nor a purely motor territory, but a graded cross-network transition linking linguistic representations to action systems.

This account differs from treating network integration as either overlap between two modules or communication through a functionally undifferentiated hub. The two ends of 55b were not distinguished by connectivity alone: their network alignments were mirrored by representational content, task preference, and a crossed pattern of anatomical connectivity estimates. This convergence implies an organized transition in which the language-facing end retains stronger access to linguistic and category information, while the SCAN-facing end is progressively biased toward action formulation and articulatory structure. The median split used here provides an operational comparison between the ends of this axis, but the underlying result is most naturally interpreted as a continuum rather than two new cortical modules. Such a local gradient offers a simple way to combine segregation with integration: different computations can be emphasized at opposite ends while neighboring circuitry allows information to be reformatted across the territory.

The gradient also reconciles previously divergent accounts of area 55b. Dorsal frontal language models and neurosurgical studies have emphasized contributions to speech planning, syllabic sequencing, and articulatory control ^13,14,17,28–30,42,43^, whereas other work has placed the same territory within a broader language or semantic architecture ^32–36^. If 55b is treated as a single functional unit, these assignments appear incompatible. The present results show why both can be partly correct. The posterior, SCAN-aligned end was more strongly engaged by production and carried stronger articulatory-feature encoding, whereas the anterior, language-aligned end carried stronger semantic-category information. Area-level labels can therefore obscure a computational transition within the territory, particularly when inter-individual variability and group averaging blur its internal topography ^44,45^.

The relationship with SCAN is especially informative. SCAN regions interrupt the classical effector map and have been proposed to coordinate goals, bodily state, and multi-effector action plans rather than execute a single movement ^19–21^. Posterior 55b displayed the profile expected of an association-action territory: it was more strongly connected with SCAN, responded preferentially during production across speaking and writing, and carried stronger articulatory-feature encoding. This pattern places posterior 55b between high-level linguistic content and the effector-specific cortices that implement speech and writing. It also extends the relevance of SCAN beyond conventional movement paradigms to highly learned symbolic actions. Conversely, the preferential alignment of anterior 55b with the language network indicates that the transition begins within circuitry retaining access to linguistic representations rather than at the border of primary motor cortex.

Task-dependent coupling provides a dynamic complement to this stable topography. Relative to comprehension, production increased model-estimated outgoing modulation from 55b toward both laryngeal–orofacial and hand motor cortices and toward frontal language regions, while auditory and fusiform pathways showed the opposite task preference. The pattern is therefore inconsistent with a uniform increase in all 55b outputs during production. Instead, it suggests that a stable cross-network gradient can be flexibly weighted according to the current direction of processing: sensory and form-related systems are emphasized when linguistic signals are interpreted, whereas frontal and motor systems are emphasized when content is converted into action. Because the DCM was deliberately sparse and centred on 55b, these results provide evidence for task-dependent reconfiguration within the specified model, not proof that 55b is the unique causal source of the observed interactions ^40^.

The position of 55b within broader cortical hierarchies connects the local finding to a general principle of cortical organization. Across evolutionary expansion, myeloarchitecture, gene expression, aerobic glycolysis, meta-analytic function, and a composite sensory-to-association axis, the 55b parcel lay between sensorimotor-adjacent and association-adjacent cortex. This convergence is consistent with the idea that local network transitions are constrained by macroscale biological gradients ^1,2,4^. Importantly, the hierarchy analysis establishes the regional position of 55b; it does not by itself demonstrate a vertex-wise correspondence between each hierarchy map and the internal functional gradient. The two levels of evidence are complementary: intrinsic connectivity reveals the organization within 55b, while the hierarchy maps place that territory within a broader sensory-to-association landscape.

Language is a particularly clear instance of a more general cognition-to-action problem. Speaking and writing require relatively stable content to be converted into rapidly changing commands for different effectors ^46–49^; comprehension requires information to travel in the complementary direction from sensory forms toward meaning ^7,50–54^. A local gradient between a cognitive network and an action network provides an architecture capable of supporting both directions without requiring a dedicated module for every input and output format. Similar transition zones may support other flexible transformations, such as converting memory into action plans or linking perceptual decisions to coordinated behaviour. Testing that possibility will require identifying comparable within-territory gradients at other network boundaries and determining whether their topology predicts task-dependent communication.

Several limitations define the next steps. First, the current evidence is correlational. Individually targeted stimulation, intracranial recording, or precision lesion-symptom mapping will be needed to establish whether perturbing different positions along the 55b gradient produces dissociable deficits in linguistic representation and action formulation ^55–57^. Second, the production tasks were picture-cued whereas the comprehension tasks used spoken or written words. Cross-modal analyses reduce dependence on a single sensory or motor format but do not fully remove the picture-versus-word distinction; cross-format semantic generalization and models that partial out visual, orthographic, phonological, and articulatory features would provide a stronger test of representational content. Third, diffusion tractography and hypothesis-constrained DCM provide indirect estimates of anatomical and directed coupling, respectively. Their convergence with local functional gradients is informative, but neither method establishes monosynaptic pathways or causal direction. Finally, task-based subregional effects were obtained in native Chinese speakers. Replication across languages, writing systems, and naturalistic communication will clarify which aspects of the gradient are language-general.

In conclusion, area 55b contains a local anterior-posterior gradient that differentially links the language network with SCAN. Its language-facing end carries stronger semantic-category information, whereas its action-facing end carries stronger production-related and articulatory information; this functional dissociation is mirrored by white-matter connectivity and complemented by task-dependent coupling to effector systems. By locating these differences within a small cortical territory embedded in a broader sensory-to-association transition, the findings move beyond assigning 55b to a single function. They illustrate a general organizational principle by which local cortical gradients can bridge large-scale cognitive and action networks.

## Methods

### Datasets overview

This study used three neuroimaging datasets: a multimodal word-processing dataset collected by our team at the Institute of Psychology, Chinese Academy of Sciences (CAS); a published multimodal word dataset from Peking University ^37^; and a public resting-state fMRI dataset from the Human Connectome Project (HCP) ^39^. The CAS multimodal word-processing dataset served as the primary dataset for analyses. The Peking University dataset ^37^ was used to assess the robustness and cross-dataset generalizability of our findings. Resting-state fMRI data in the HCP S900 release were used to characterize local functional connectivity gradients.

### CAS multimodal word-processing dataset

#### Participants

Thirty-three healthy adults took part in this experiment. Three participants did not complete all experimental sessions and were excluded, leaving a final sample of thirty participants (16 women; mean age ± SD, 22.67 ± 2.98 years; age range, 18–34 years). All participants were right-handed native Chinese speakers with normal hearing and normal or corrected-to-normal vision. All participants provided written informed consent before participation. This study was approved by the Ethics Committee of the Institute of Psychology, Chinese Academy of Sciences (H21077), and was conducted in accordance with the Declaration of Helsinki. Participants received monetary compensation after completing the study.

#### Tasks and procedures

The CAS multimodal word-processing dataset comprised four word-level language processing tasks, including two comprehension tasks (listening and reading) and two production tasks (naming and writing). Each task included an experimental condition designed to probe lexical processing in a specific input or output modality, together with a modality-matched control condition that preserved basic sensory or motor demands while minimizing lexical and semantic content, thereby providing a baseline for isolating lexical-semantic processing. All tasks were implemented in a block design with experimental and matched control blocks. To improve the reliability of within-subject activation estimates (see **Supplementary Methods, Results** and Fig. 2), each participant completed three scanning sessions separated by at least three days, ensuring independent sampling of task-related responses. Each session comprised four runs, one for each task. The procedure and timing of each task are shown in **Supplementary Fig. 1**.

All four word-level tasks used the same set of 41 disyllabic Chinese words. 16 words were grouped into 4 semantic categories — household appliances, places, mammals and vehicles — with 4 words in each category; each category formed one semantic block. The remaining 25 words were grouped into 5 phonological sets according to the onset syllable of the first character (shu, shi, xiang, luo and jing), with 5 words in each set; each set formed one phonological block. Each task included 4 semantic blocks, 5 phonological blocks and 6 modality-matched control blocks. Each semantic block contained 4 items and each phonological block contained 5 items. Among the 6 control blocks, 3 blocks each contained 4 items to match the semantic blocks, and the other 3 blocks each contained 5 items to match the phonological blocks. The full set of stimuli was presented in **Supplementary Table 3**. We counted word frequency, first-syllable frequency, character frequency and stroke count. For the production tasks, we selected object pictures corresponding to the target words from standardized picture databases with high naming agreement ^58,59^.

#### Listening task

In the listening task, participants listened to spoken words in the experimental blocks and reported at the end of each block whether any animal-related words had appeared. In the control blocks, they listened to acoustically matched reversed-speech stimuli and reported whether they had heard any meaningful speech ^37,60^. The spoken-word stimuli were generated using female voice text-to-speech synthesis and the control stimuli were created by applying full time reversal to each audio file, producing unintelligible stimuli that preserved low-level acoustic properties. Each block began with a 200-ms fixation cross and was followed by a semantic, phonological or control block. Within each block, each item was presented for 2,000 ms with a fixed 250 ms interstimulus interval. After each block, a probe question appeared for 2,500 ms. Blocks were separated by fixation intervals of 9–12 s (mean, 11 s), yielding a total run duration of 5 min 58 s.

#### Reading task

In the reading task, participants viewed Chinese disyllabic words during the experimental blocks and judged at the end of each block whether any animal terms had appeared. During the control blocks, they viewed Korean Hangul strings and judged whether any Chinese characters had appeared. Korean Hangul strings were used as a visual control to preserve low-level orthographic input while minimizing access to lexical-semantic content ^61,62^. Before the experiment, we confirmed that participants could not read or identify Hangul characters. Each block began with a 200-ms fixation cross. Stimuli were presented for 2,000 ms with a fixed 250-ms interstimulus interval, followed by a 2,500-ms response period at the end of each block. Blocks were separated by fixation intervals of 9–12 s, yielding a total run duration of 5 min 58 s.

#### Naming task

In the naming task, participants viewed nameable object pictures and named each object aloud during the experimental blocks, whereas during the control blocks they viewed scrambled versions of the same pictures and produced a fixed utterance (“1, 2″). Spoken responses were recorded during scanning using an external microphone. Before scanning, participants completed a naming practice session to ensure consistent labels for the object pictures. The scrambled images were created by randomly rotating pixel blocks to preserve low-level visual properties, such as luminance and contrast, while disrupting recognizable structure ^60^. Each block began with a 200-ms fixation cross, after which each stimulus was presented for 2,500 ms with a fixed 250-ms interstimulus interval. Blocks were separated by fixation intervals of 9–12 s, yielding a total run duration of 5 min 55 s.

#### Writing Task

In the writing task, participants viewed object pictures and wrote the corresponding Chinese disyllabic words in the experimental blocks and viewed geometric line drawings adapted from previous studies ^63,64^ and copied the figures in the control blocks. Responses were recorded using a custom fMRI-compatible tablet equipped with a touch-sensitive surface, a force-sensitive stylus, and an adjustable support frame. The support frame was individually adjusted to provide stable wrist or forearm support, thereby reducing fatigue during scanning. Real-time ink feedback was provided to allow participants to monitor their written or drawn output during the task. Participants were instructed to write each character stroke by stroke while minimizing upper-arm and forearm movements to reduce head motion. Before the formal experiment, all participants completed in-scanner practice with the tablet by writing five example words. Each block began with a 200-ms fixation cross, followed by image presentation, with each item displayed for 1.5 s. After each image appeared, participants had 3–5 s to respond, depending on the stroke count of the target characters in the experimental condition. After each line drawing appeared, participants had 3 s to respond. Blocks were separated by fixation intervals of 9–12 s, yielding a total run duration of 11 min 46 s.

### MRI data acquisition and preprocessing

#### MRI data acquisition

MRI data were acquired using a 3.0-T Siemens Magnetom Prisma scanner at the MRI Center of the Institute of Biophysics, Chinese Academy of Sciences. High-resolution structural images were acquired using a T1-weighted magnetization-prepared rapid acquisition gradient-echo (MPRAGE) sequence (repetition time (TR) = 2200 ms; echo time (TE) = 3.49 ms; 192 sagittal slices; field of view (FOV) = 256 mm; voxel size = 1×1×1 mm).

Task and resting-state functional images were acquired using a multiband echo-planar imaging (EPI) sequence (multiband factor = 2; TR = 1500 ms; TE = 30 ms; 40 axial slices; FOV = 192 mm; voxel size = 3×3×3 mm). Task fMRI was acquired throughout the full duration of each task run, with an additional 9-s dummy period at the start of each run to allow for magnetization stabilization. Resting-state scans were acquired over 15 min in two runs of 8 min and 7 min. Diffusion-weighted images were collected using a high–angular-resolution diffusion imaging (HARDI) protocol (TR = 4000 ms; TE = 79 ms; voxel size = 1.5×1.5×1.5 mm; field of view = 192 mm). The sequence included 64 diffusion directions at *b* = 1000 s/mm^2^ and 64 directions at *b* = 2000 s/mm^2^, and five non-diffusion-weighted (*b* = 0 s/mm^2^) volumes. Structural, resting-state and diffusion scans were acquired on separate days from the task fMRI sessions to minimize fatigue.

#### Preprocessing of MRI data

Structural, task-based and resting-state fMRI data were preprocessed using *fMRIPrep* ^65,66^ followed by *XCP-D* ^67^. Structural T1-weighted images underwent intensity non-uniformity correction ^68^, skull stripping, tissue segmentation, and cortical surface reconstruction using FreeSurfer’s recon-all pipeline ^69^. fMRI data underwent susceptibility distortion correction using field maps, co-registration of the BOLD reference image to each participant’s T1-weighted anatomical image with FreeSurfer’s bbregister, head-motion correction with FSL’s mcflirt, slice-timing correction with AFNI’s 3dTshift, and cortical surface reconstruction using FreeSurfer’s recon-all pipeline. Data were then projected to grayordinates by generating HCP fsLR 64k cortical meshes with wb_command and resampling BOLD time series to the cortical surface and subcortical grey-matter structures ^39^. Finally, time series were despiked and temporally detrended using AFNI’s 3dDespike to remove transient outliers and linear or quadratic drifts ^70^. Resting-state fMRI data underwent the same preprocessing steps, followed by additional denoising procedures. These included 36-parameter confound regression, incorporating 24 motion parameters together with mean white-matter, cerebrospinal fluid and global signals, and band-pass filtering (0.01–0.08 Hz) to reduce high-frequency physiological noise and low-frequency scanner drift ^71–74^.

High-angular-resolution diffusion-weighted imaging (HARDI) data were preprocessed using QSIPrep and reconstructed for tractography using the QSIRecon MRtrix3 workflow ^75^. The T1-weighted anatomical reference image was first reoriented into AC–PC alignment using a 6-degree-of-freedom transform estimated from affine registration to the MNI152NLin2009cAsym template. Nonlinear registration to the same template was then performed using symmetric normalization in ANTs. Brain extraction was performed with SynthStrip ^76^, and tissue segmentation was obtained using SynthSeg from FreeSurfer 7.3.1^77^.

Diffusion preprocessing followed the standard QSIPrep workflow. Raw diffusion images were denoised using Marchenko–Pastur principal component analysis as implemented in MRtrix3 dwidenoise, with an automatically determined five-voxel window ^78,79^. Gibbs ringing artifacts were then removed using MRtrix3 *mrdegibbs* ^80^. Volumes with b-values below 100 s/mm^2^ were treated as b0 images. When multiple DWI sequences were acquired, the mean intensity of each diffusion series was normalized so that the mean b0 intensity was matched across sequences. Susceptibility-related and eddy-current distortions, as well as head motion, were corrected using FSL eddy ^81^. Eddy was run with outlier replacement, post-correction shell alignment, a q-space smoothing factor of 10, five iterations, and 1000 voxels for hyperparameter estimation ^82^. A quadratic first-level model and a linear second-level model were used to model eddy-current-related spatial distortions. After correction and resampling, B1 field inhomogeneity was corrected using MRtrix3 *dwibiascorrect* with the N4 algorithm ^68^. The preprocessed DWI series was resampled to AC–PC space at 1.5-mm isotropic resolution. Framewise displacement and slice-wise cross-correlation metrics were computed for quality control.

Whole-brain tractography used the QSIRecon mrtrix_multishell_msmt_ACT-hsvs workflow. Multi-tissue fibre orientation distributions were estimated from multi-shell HARDI data using multi-shell multi-tissue constrained spherical deconvolution, with separate response functions for white matter, grey matter, and cerebrospinal fluid ^76^. Probabilistic streamlines were generated using second-order integration over fibre orientation distributions (iFOD2) in MRtrix3 ^83^. Anatomically constrained tractography used tissue boundaries from T1-weighted segmentation and the hybrid surface–volume segmentation procedure of the ACT-HSVS workflow ^84^. SIFT2 weights were estimated for the whole-brain tractogram ^85^, although the regional connectivity analysis reported below used normalized retained-streamline counts rather than the summed SIFT2 weights. Whole-brain tractograms were visually inspected before region-to-region tract extraction. For task-fMRI quality control, participants would have been excluded if any run exceeded 3 mm translation, 3° rotation, or mean framewise displacement of 0.2 mm; no CAS participant met these criteria.

### Peking University Dataset

#### Participants

We used a separate multimodal word-processing dataset collected at Peking University^37^ to evaluate the robustness and generalizability of our findings. The original sample comprised 101 healthy native Chinese speakers (50 women; mean age ± SD, 23.0 ± 2.2 years). One male participant was excluded due to excessive head motion (> 2.5 mm), yielding a final sample of 100 right-handed participants. All participants reported normal hearing, normal or corrected-to-normal vision, and no history of neurological or language disorders. Written informed consent was obtained from all participants, and the study was approved by the Institutional Review Board at Peking University.

#### Tasks

Like the CAS multimodal word-processing dataset, this dataset comprised four word-level language tasks: two comprehension tasks (listening and reading) and two production tasks (naming and writing), each with experimental and control blocks. Unlike the CAS multimodal word-processing dataset, however, the lexical items were not matched across tasks, such that each task used a distinct word set. In addition, the control contrasts and task procedures differed from those used in the corresponding CAS tasks. In the listening task, participants heard spoken Chinese nouns during the experimental blocks and judged whether each referred to a living creature; during the control blocks, they heard time-reversed speech and judged whether the speaker was male or female. In the reading task, participants viewed written Chinese nouns during the experimental blocks and judged whether each referred to a living creature, whereas during the control blocks they viewed written Chinese words and judged the number of characters in each item. In the naming task, participants viewed line drawings of common objects and named each picture aloud during the experimental blocks, whereas during the control blocks they viewed matched syllable strings and repeated them aloud. In the writing task, participants viewed object pictures and wrote the corresponding words on an MRI-compatible tablet during the experimental blocks, whereas during the control blocks they copied visually presented Chinese characters (or control symbols) with their right hand. Full details are provided in Qin et al. (2021) ^37^.

#### Data acquisition and preprocessing

MRI data were acquired on a 3T Siemens Prisma scanner (Siemens Healthineers) with a 20-channel head coil. A high-resolution T1-weighted structural image was obtained using an MPRAGE sequence (TR = 2530 ms; TE = 2.98 ms; flip angle = 7°, 1 mm isotropic voxels) for anatomical reference and surface reconstruction. Task and resting-state functional images were acquired using a T2*-weighted EPI sequence with 33 contiguous axial slices covering the whole brain (TR = 2000 ms, TE = 30 ms, flip angle = 90°, matrix size = 64 64, in-plane resolution = 3.5× 3.5 mm, and slice thickness = 4.2 mm). The resting state run lasted 6 min. These data were preprocessed using the same *fMRIPrep* and *XCP-D* workflow described above.

### Human Connectome Project dataset

#### Participants

To characterize large-scale intrinsic functional gradients, we analyzed resting-state fMRI data from 245 neurologically healthy adults (130 men, 115 women) from the HCP S900 release ^39^. Participants were aged 23 to 35 years (mean ± SD, 28.21 ± 3.67 years). Participants were included based on HCP motion-quality metrics. Individuals were excluded if they exceeded 1.5 times the interquartile range on at least two of four summary motion measures derived from relative root mean square displacement across resting-state runs. Runs were further excluded if more than 25% of frames exceeded 0.2 mm frame-wise displacement ^86–88^. Three participants with incomplete resting-state time series were also removed. Only unrelated individuals who completed all four resting-state runs were retained for analysis.

#### MRI data acquisition and preprocessing

MRI acquisition protocols for the HCP have been described elsewhere ^38,39^. All data were collected on a customized 3-T Siemens Connectome scanner with a 100 mT/m SC72 gradient system and a 32-channel head coil. We used only the T1-weighted structural images and resting-state fMRI data in the present study.

High-resolution T1-weighted structural images were acquired using an MPRAGE sequence with 0.7-mm isotropic resolution. Resting-state fMRI data consisted of four runs, each lasting 14 min 33 s, acquired using a multiband echo-planar imaging sequence (TR = 720 ms, 2-mm isotropic) optimized for high temporal and spatial resolution.

#### Preprocessing

Resting-state fMRI data from the HCP S900 release were processed using the HCP minimal preprocessing pipelines ^39^. Structural images were corrected for spatial distortions, registered to MNI152 space using nonlinear volume-based alignment, and processed via FreeSurfer v5.3 to reconstruct cortical surfaces. Functional images underwent distortion correction, motion correction and registration to the T1-weighted image, followed by ribbon-constrained volume-to-surface mapping to generate CIFTI grayordinate data. Subcortical voxels were assigned to their corresponding structures, and cortical and subcortical data were combined in standard CIFTI grayordinate space. Surface smoothing (2-mm FWHM) was applied to reduce mixing signals across sulcal banks and areal boundaries. Resting-state fMRI data were further denoised using spatial ICA+FIX for spatially specific noise and temporal ICA ^22,89^.

### First-level activation analysis

#### CAS multimodal word-processing dataset

We estimated single-block responses for each participant, session and task for use in subsequent univariate analyses, multivariate pattern analyses, semantic decoding and gradient-based functional characterization. Single-block responses were estimated using *GLMsingle*, which improves block-wise fMRI response estimation through spatially specific hemodynamic response function selection, data-driven denoising and fractional ridge regression within a unified general linear modelling framework, thereby yielding more reliable block-wise beta estimates ^90^. For each block, onset times and durations were entered into the design matrix, with six rigid-body motion parameters included as nuisance regressors. Block-wise beta estimates were computed in CIFTI grayordinate space.

#### Peking University dataset

Because the Peking University dataset used different word items across tasks, we restricted analyses to task-wise univariate contrasts between word blocks and their matched control blocks. We estimated block-level activation maps using a standard first-level general linear model implemented in Nilearn ^91^. For each participant and task, BOLD time series were modelled with regressors for word and control blocks, convolved with a Glover hemodynamic response function. Polynomial drift terms (order 3) and six rigid-body motion parameters were included as nuisance regressors. Beta estimates were computed in CIFTI grayordinate space. We obtained word versus control statistical maps by computing t-contrast Z-maps for each task using Nilearn’s contrast estimation routines. These maps were used for univariate activation replication and gradient-based functional subregion segmentation analysis.

#### Individual-specific brain parcellation

To improve cross-participant correspondence while accommodating individual variability, all task analyses used individual-specific functional parcellations ^23,24,45^. We estimated 900 cortical parcels for each participant with the gradient-informed multi-session hierarchical Bayesian model (gMS-HBM) ^23^, applied to the participant’s 15-min resting-state scan. Whole-brain connectivity gradients were used to construct participant-specific features in a high-dimensional gradient space. Parcellation was initialized from a Schaefer-900 group prior pretrained on 40 Human Connectome Project participants, and subject-specific parcels were estimated using gMS-HBM with w = 30, c = 30, and β = 50, following Kong et al. ^23^ (see **Supplementary Methods**). Activation modelling, MVPA, and DCM volume-of-interest definition used these individual-specific parcels.

For the Peking University dataset, the 6-min resting-state scan was insufficient to support stable estimation of individual-specific parcellations. We therefore concatenated each participant’s resting-state time series with residual BOLD time series from the task runs after regressing out task effects, and used this combined intrinsic signal to compute individual-specific parcellations ^45,92–95^. Full procedural details are provided in the **Supplementary Methods** (*Individual-specific brain parcellation based on gMS-HBM*).

#### Multimodal lexical-network mapping and conjunction analysis

To identify regions consistently engaged across listening, reading, naming and writing, we performed parcel-wise group-level analyses on activation estimates derived from individual-specific parcellations, followed by conjunction mapping across tasks (workflow in Fig. 1a). For each participant, session and task, we averaged block-wise activation estimates within each parcel separately for the experimental and control conditions. Because the experimental condition contained more blocks than the control condition, we matched the number of experimental blocks to the number of control blocks within each session when computing condition averages, to ensure comparable signal-to-noise ratios between conditions.

For each task (listening, naming, reading and writing), we fitted a linear mixed-effects model separately for each parcel, with condition (experimental = 1, control = 0) as the fixed effect of interest, to estimate the group-level activation difference between word and control blocks. Specifically, for each participant s, session k, and parcel i, the activation estimate $y_{i,s,k}$ was modeled as:

$$y_{i,s,k}=\beta_{0,i}+\beta_{1,i}X_{s,k}+u_{s,i}+v_{k,i}+\varepsilon_{i,s,k},$$

where $\beta_{0,i}$ was the parcel-specific intercept, $X_{s,k}$ was a binary indicator coding condition (experimental condition = 1, control condition = 0), $\beta_{1,i}$ was the parcel-wise fixed effect indexing the activation difference between the experimental and control conditions, $u_{s,i}\sim 𝒩\left(0,\sigma_{u,i}^{2}\right)$ was the participant-specific random intercept, $v_{k,i}\sim 𝒩\left(0,\sigma_{v,i}^{2}\right)$ was the session-specific random intercept, and $\varepsilon_{i,s,k}\sim 𝒩\left(0,\sigma_{\varepsilon ,i}^{2}\right)$ was the residual error term. We fitted the models in R 4.4.0 using the *lmerTest* package. Across the 900 parcels, p values were corrected for multiple comparisons using false discovery rate (FDR) correction at *q* < 0.05.

To identify regions consistently engaged across listening, reading, naming and writing, we applied the minimum-T conjunction method in SPM (https://www.fil.ion.ucl.ac.uk/spm/) to the group-level activation maps from the four-word tasks (Fig. 1a). To further assess the robustness of these findings, we repeated the same mixed-effects analysis at the vertex level and generated vertex-wise activation and conjunction maps.

#### Analytical framework for characterizing a cross-network transition

The conjunction analysis first defined a modality-invariant lexical candidate network (Fig. 1a). The remaining analyses follow the order used in the Results and are summarized in Fig. 1b, c. We estimated the internal connectivity gradient of area 55b and its large-scale alignment with the language network and SCAN, and then tested whether gradient position predicted task responses, semantic-category information, articulatory-feature encoding, and tractography-derived connectivity. We next quantified comprehension–production differentiation across sensory and motor formats and task-domain modulation of semantic-category information, followed by task-dependent model-estimated directed coupling on 55b-centred edges. Finally, we situated 55b and comparison regions within local transitions across six macroscale cortical hierarchy maps. This sequence treats the analyses as convergent characterization of a language-to-SCAN transition rather than as criteria for cortical exclusivity.

#### Internal gradient and large-scale network alignment

We asked whether area 55b contained an internal connectivity gradient that differentially aligned with the language network and SCAN (Fig. 1b). We estimated the dominant local connectivity gradient and used a median split to compare its anterior and posterior ends in large-scale network alignment. The split provided an operational comparison, whereas the underlying organization was treated as continuous. The gradient-defined sectors were then carried forward to the functional, representational, and white-matter analyses described in the next Methods module. The same local-gradient procedure was applied to SFL as a specificity analysis reported in the Supplementary Results.

#### Local functional connectivity gradient analysis

Area 55b was the primary local-gradient target because multimodal lexical mapping placed it at the junction of the language network and SCAN. SFL was analyzed in parallel as a regional specificity comparison. The individualized Schaefer–Kong parcels were too small for stable within-region subdivision, so each region was mapped to the corresponding Glasser 360 parcel. For area 55b, 93% of vertices overlapped with Glasser area 55b, and the full Glasser-defined territory was used for gradient analysis; SFL was handled analogously. Gradient estimation was performed separately in each dataset. In the Peking University dataset, the 6-min resting-state scan was concatenated with 15 min of residual task-fMRI time series after task-effect regression to increase the intrinsic time series available for estimation.

Within each participant, we computed a whole-cortex functional connectivity profile for every vertex in the region by correlating its resting-state time series with the time series of all cortical vertices in fs_LR 32k space (59,412 vertices). This yielded a vertex-by-cortex functional connectivity matrix, which was then Fisher z-transformed. These matrices were averaged across participants to obtain a group-level functional connectivity matrix for the region. Cosine similarity was then computed between vertices within the region to generate a vertex-by-vertex affinity matrix. Diffusion embedding, as implemented in BrainSpace ^96^, was applied to this affinity matrix to derive connectivity gradients. The first gradient, which captured the dominant mode of variation in local connectivity organization, was taken as the group-level principal gradient ^4,96,97^.

Subject-specific gradients were then estimated from each participant’s Fisher z-transformed vertex-by-cortex functional connectivity matrix using the same procedure. Cosine similarity was used to construct an individual affinity matrix, diffusion embedding was applied to derive the individual principal gradient, and each individual gradient was aligned to the corresponding group-level principal gradient using Procrustes alignment ^96^. For each participant, the aligned principal gradient assigned a gradient value for every vertex in the region. Vertices were then subdivided into high-gradient and low-gradient subregions using a median split of these values.

#### Large-scale network alignment of gradient-defined subregions

To determine the large-scale network alignment of the gradient-defined subregions, we first computed whole-cortex functional connectivity maps for each subregion. For each participant, the mean BOLD time series was extracted from each subregion and correlated with the time series of all cortical vertices, followed by Fisher z transformation. These seed-to-cortex connectivity maps were then averaged across participants and thresholded using FDR correction at *q* < 0.05.

We next quantified the spatial overlap between each thresholded connectivity map and several canonical network templates, including the 17-network cortical parcellation ^23,98^, a fronto-temporal language network ^99^, a semantic control network ^100^, the SCAN ^19^ and the action mode network ^21^. Additional language- and action-related systems were included to assess whether the overlap patterns were specific to established language networks or extended to recently proposed action-related systems. All networks were binarized in surface space, and overlap was quantified using the Dice coefficient. For each subregion, the template with the highest Dice coefficient was taken as its best-matching large-scale network.

### Functional and structural specialization along the 55b gradient

#### Relative responses during comprehension and production tasks

To determine whether gradient-defined subregions differed in their engagement during word comprehension and production, we assessed task responses using two complementary analyses: univariate activation contrasts and multivariate pattern classification. Both analyses were performed separately within the high-gradient and low-gradient subregions of each candidate region. We predicted that sensorimotor-biased subregions would show stronger production-dominant responses and stronger discrimination between comprehension and production than association-biased subregions.

#### (1) Subregional production bias in univariate activation

For each participant, block-wise activation estimates were averaged separately across the comprehension tasks (listening and reading) and the production tasks (naming and writing) for each gradient-defined subregion. A production-bias index was then computed for each subregion as the mean activation during production minus the mean activation during comprehension.

The primary analysis tested whether production bias differed between the two subregions within each candidate region. Paired-sample t-tests were used to compare the task-bias index between the high-gradient and low-gradient subregions across participants. Follow-up one-sample t-tests were then performed separately for each subregion to determine whether its production-bias index was significantly greater than zero, indicating stronger responses during production than comprehension. P values from all between-subregion and within-subregion tests across candidate regions were corrected for multiple comparisons using FDR correction at *q* < 0.05.

#### Subregional decoding of the comprehension–production distinction

We next tested whether multivariate activation patterns within each gradient-defined subregion distinguished comprehension from production. To do so, we repeated the cross-modal multivariate pattern classification analysis described above separately within each subregion, using only the vertices belonging to that subregion as features.

For each participant and subregion, block-wise beta estimates from the experimental word blocks of the four word-level tasks were entered into the same cross-modal classification framework described above. Decoding accuracies were averaged across the four comprehension–production train–test combinations to yield the mean comprehension–production decoding accuracy for each participant and subregion. The primary analysis tested whether comprehension–production decoding accuracy differed between the two subregions within each candidate region. Paired-sample t-tests were used to compare decoding accuracy between the high-gradient and low-gradient subregions across participants. Follow-up one-sample t-tests were then performed separately for each subregion to determine whether decoding accuracy exceeded chance (0.5). P values from all between-subregion and within-subregion tests across candidate regions were corrected for multiple comparisons using FDR correction at *q* < 0.05.

#### Representational content of gradient-defined subregions

To test whether gradient-defined subregions differed in representational content along the local connectivity gradient, we quantified semantic category decoding and articulatory-feature encoding within each subregion. Specifically, we asked whether association-biased subregions showed stronger semantic category decoding, whereas sensorimotor-biased subregions showed stronger articulatory-feature encoding. To address this question, we performed subregional semantic category decoding and subregional articulatory-feature encoding, and compared the resulting indices between the high-gradient and low-gradient subregions of each candidate region.

#### Subregional semantic-category decoding

To assess semantic category representation, we repeated the semantic decoding analysis described above within each gradient-defined subregion, restricting the feature space to the vertices belonging to that subregion. Semantic category decoding was performed using the same pairwise classification procedure and nested LOSO framework described in the preceding semantic-category analysis. For each participant, task and subregion, decoding accuracies were averaged across the six category pairs to yield a task-specific semantic decoding score. These scores were then averaged across the four word-level tasks to obtain a single semantic decoding index for each participant and subregion. The primary analysis tested whether this semantic decoding index differed between the high-gradient and low-gradient subregions within each candidate region. Paired-sample t-tests were used for this comparison. P values from these between-subregion tests across candidate regions were corrected for multiple comparisons using FDR correction at q < 0.05.

#### Subregional articulatory-feature encoding

To assess articulatory-feature encoding, we tested whether the high-gradient and low-gradient subregions within each candidate region differed in how strongly their activity was explained by articulatory features during word processing. This analysis focused on the listening, naming and reading tasks; the writing task was excluded because continuous hand movements disrupted the temporal correspondence between linguistic events and BOLD responses.

For each word, articulatory features were represented in a 28-dimensional binary feature space, with each dimension corresponding to one articulatory feature used in Chinese. Feature vectors were summed across syllables to obtain a single 28-dimensional articulatory vector for each word. Two sets of predictors were then constructed from the event timings: an articulatory regressor set containing word onsets, word durations and the corresponding articulatory vectors, and a word-onset-only regressor set containing the same word onset and word duration information but no articulatory features. To align these predictors with the BOLD signal, both regressor sets were resampled to the fMRI sampling grid using Lanczos interpolation and expanded into finite impulse response (FIR) design matrices with six delays, corresponding to six successive TRs and spanning 9 s ^101–104^.

For each participant and subregion, continuous BOLD time series were extracted from block onset to block offset for the listening, naming and reading tasks, and the resulting block-level time series were concatenated across tasks to form a single response vector ^105^. This procedure ensured that successful model fits reflected articulatory information that generalized across tasks rather than task-specific variance.

Two ridge-regression encoding models were then fitted separately for each subregion. The full model included the delayed articulatory regressors together with the delayed word-onset-only regressors, whereas the control model only included the delayed word-onset-only regressors. The word-onset-only model controlled for the temporal effects of word occurrence, such that the difference between the two models reflected variance uniquely attributable to articulatory features. Model fitting used nested 10-fold cross-validation repeated 100 times ^103,104^.

Prediction performance was quantified as the cross-validated correlation between predicted and observed BOLD responses, averaged across the 100 repetitions. These correlations were then converted into an articulatory-feature encoding index,

$$G=\frac{R_{\text{full}}^{2}\;-\;R_{\text{onset}}^{2}}{R_{\text{full}}^{2}\;+\;R_{\text{onset}}^{2}},$$

which captured the relative gain in explained variance attributable to the articulatory regressors beyond the word onset-only regressors. The primary analysis tested whether this encoding index differed between the high-gradient and low-gradient subregions within each candidate region. Paired-sample t-tests were used for this comparison. P values from these between-subregion tests across candidate regions were corrected for multiple comparisons using FDR correction at *q* < 0.05.

#### White-matter connectivity of anterior and posterior area 55b

We tested whether the anterior–posterior functional organization of area 55b was mirrored by tractography-derived connectivity with two large-scale systems: the language network (LAN) ^99^ and the somato-cognitive action network (SCAN) ^19^. We predicted that anterior 55b, which carried stronger semantic-category information, would show stronger normalized streamline-count connectivity with LAN, whereas posterior 55b, which showed stronger production bias and articulatory-feature encoding, would show stronger connectivity with SCAN.

For each participant, tracts were extracted from the whole-brain tractogram using MRtrix3 tckedit ^78^. Anterior and posterior 55b seed masks were defined from the participant-specific subdivision of area 55b and transformed into the participant’s diffusion space. LAN and SCAN target masks were derived from the corresponding network masks and mapped into the same diffusion space using individual anatomical and surface registrations. For each seed–target pair, we retained streamlines whose endpoints intersected both the 55b seed mask and the target network mask. Exclusion masks were applied to remove streamlines entering the right hemisphere or left-hemisphere cortical regions outside the corresponding seed–target pair. Tract-density maps were generated from the retained streamlines and visually inspected for quality control.

We quantified connection strength using a normalized streamline-count measure. For each seed-target tract, we counted the number of retained streamlines and normalized this value by the geometric mean of the seed and target mask sizes:

$$\text{Connectivity}=\frac{N_{\text{streamlines}}}{\sqrt{V_{\text{seed}}\;\times \;V_{\text{target}}}},$$

where $N_{\text{streamlines}}$ denotes the number of retained streamlines, and $V_{\text{seed}}$ and $V_{\text{target}}$ denote the number of voxels in the seed and target masks, respectively. This normalization reduced the influence of inter-individual differences in ROI size on the estimated connection strength ^75^. For each participant, normalized connectivity was computed separately for four seed–target pairs: anterior 55b–LAN, anterior 55b–SCAN, posterior 55b–LAN and posterior 55b–SCAN.

We tested whether anterior and posterior 55b showed dissociable structural connectivity profiles using a 2 × 2 repeated-measures ANOVA, with seed subregion (anterior 55b, posterior 55b) and network (LAN, SCAN) as within-participant factors and normalized connectivity as the dependent variable. The key effect of interest was the seed subregion × network interaction, which tested whether the anterior–posterior difference in connection strength depended on the target network. When this interaction was significant, planned simple-effects analyses compared anterior and posterior 55b connectivity separately within LAN and SCAN. Statistical inference was restricted to participants with complete connectivity estimates across all four seed-by-network conditions. Generalized η^2^ was reported for repeated-measures ANOVA effects, and Cohen’s *d* was reported for paired simple-effects comparisons.

#### Comprehension–production differentiation across sensory and motor formats

We assessed whether conjunction-defined regions distinguished word comprehension from word production using two complementary analyses: one based on univariate activation magnitude and the other on multivariate activation patterns.

#### Activation-strength analysis

For each participant and each conjunction-defined region, block-wise activation estimates were averaged separately across comprehension (listening and reading) and production (naming and writing). Within each region, production and comprehension estimates were compared using paired-sample t tests. P values were Bonferroni-corrected across the four conjunction-defined regions, matching the correction family reported in the Results.

#### Multivariate classification of the comprehension–production distinction

To test whether conjunction-defined regions encoded a modality-invariant distinction between comprehension and production, we used a cross-modal multivariate pattern classification framework in which the classifier was trained on one comprehension–production task pair and tested on another pair that preserved the same distinction but involved different input or output modalities. Four train–test combinations were used: the classifier was trained to distinguish listening from naming and tested on distinguishing reading from writing, and vice versa; it was also trained to distinguish listening from writing and tested on distinguishing reading from naming, and vice versa. As a control analysis, we tested whether decoding instead reflected orthographic grouping by distinguishing tasks involving written word forms (reading and writing) from tasks without written word forms (listening and naming). Using the same framework, the classifier was trained on the listening-versus-reading contrast and tested on the naming-versus-writing contrast, or vice versa.

For each participant, we extracted block-wise beta estimates for the experimental word blocks only and averaged them across the three sessions. This yielded a 9×Nv pattern matrix for each task, where 9 denotes the 4 semantic blocks and 5 phonological blocks, and Nv denotes the number of vertices within the region. For each train–test combination, these matrices were used to construct a classification dataset comprising 18 samples (two classes × nine blocks), labelled according to the dimension being tested.

Classification was performed using an RBF kernel-based support vector machine implemented in LIBSVM 3.25 within a nested leave-one-subject-out (LOSO) framework ^106,107^. In the inner loop, the penalty parameter C and the RBF kernel parameter γ were optimized by leave-one-subject-out cross-validation on the remaining participants using predefined candidate grids. Features were demeaned and min–max normalized using the training data, and the same scaling parameters were then applied to the held-out participant.

Decoding accuracy was defined as the proportion of correctly classified test samples (chance = 50%). For each participant and region, accuracies were averaged across the four comprehension– production train–test combinations and, separately, across the two orthographic-control combinations. Mean accuracy was tested against chance using one-tailed one-sample t tests with Bonferroni correction across regions (*P*_corrected_ < 0.05). Comprehension–production and orthographic-control accuracies were also compared using paired-sample t tests. A region was considered to carry a cross-modal comprehension–production distinction when comprehension–production accuracy exceeded chance and orthographic-control accuracy, while orthographic-control accuracy did not exceed chance. Because both production tasks were picture-cued, this criterion does not eliminate all stimulus-format differences between task domains.

#### PCA-based visualization of multivariate task representations

To visualize the representational structure underlying the multivariate pattern classification results, we projected task-specific activation patterns into a low-dimensional space using PCA. PCA was applied to the same block-wise beta estimates from the experimental word blocks used in the multivariate pattern classification analyses. For each region, block-wise activation patterns were first averaged within each task across participants, yielding four group-level task-specific activation patterns, corresponding to listening, reading, naming, and writing, in the original high-dimensional feature space. These patterns were then projected into a low-dimensional representational space to define the group-level embedding used for visualization.

To visualize individual-level representations in a common space, the same procedure was applied to subject-specific block-wise beta estimates from the experimental word blocks. For each participant and each region, block-wise activation patterns were averaged within each task to yield four task-specific activation patterns, which were then projected into a low-dimensional space. Each participant’s embedding was aligned to the group-level solution using Procrustes analysis ^4,96,97^. This alignment preserved the internal representational structure of task representations while placing them in a shared coordinate system. All analyses were implemented in MATLAB R2021a ^108^.

#### Task-domain modulation of semantic-category information

We asked whether semantic-category information differed between production and comprehension in the regions retained from the preceding analysis. Multivariate category decoding was performed separately for each word-level task; accuracy was then averaged across naming and writing to obtain a production score and across listening and reading to obtain a comprehension score. The term semantic-category information is used because the design did not test cross-format generalization between pictures and words.

#### Multivariate semantic category classification

For each participant and each region, block-wise beta estimates were extracted from the experimental word blocks. Within each task, activation patterns for each semantic category were averaged across the three sessions, yielding a 4×Nv feature matrix, where the four rows correspond to the semantic categories (household appliances, places, mammals and vehicles) and Nv denoted the number of vertices within the region. The four categories yielded six pairwise comparisons. For each category pair, multivariate classification was performed to distinguish the two category-specific activation patterns, using an RBF kernel-based support vector machine implemented in LIBSVM 3.25 within a nested LOSO framework used for the task-domain classification analysis ^106,107^. In the inner loop, the penalty parameter C and the RBF kernel parameter γ were optimized by leave-one-subject-out cross-validation on the remaining participants using predefined candidate grids. Features were demeaned and min–max normalized using the training data, and the same scaling parameters were then applied to the held-out participant.

Classification accuracy was defined as the proportion of correctly classified samples in the test set (chance = 50%). For each participant, region and task, decoding accuracies were averaged across the six category pairs to yield a task-specific semantic decoding score. These task-specific scores were then averaged across listening and reading to derive a comprehension decoding score and across naming and writing to derive a production decoding score. Within each region, production and comprehension decoding scores were compared across participants using paired-sample t-tests. To further characterize the source of any production–comprehension difference, production and comprehension decoding scores were also tested separately against chance using one-sample t-tests. P values were corrected for multiple comparisons using FDR correction at *q* < 0.05.

#### Task-dependent model-estimated directed coupling via DCM

We used DCM implemented in SPM12 to test whether language production and comprehension were associated with different model-estimated directed coupling on area 55b-centred edges. The a priori contrast asked whether production increased coupling from 55b toward frontal language-related and motor-effector regions. A sparse 55b-centred architecture was used to constrain the hypothesis space. The analysis therefore estimates task-dependent coupling within the specified model and does not establish a unique whole-network causal pathway.

The model included eight task-relevant regions spanning input, lexical and output components of the four word-processing tasks: auditory cortex, visual cortex, area 55b, anterior fusiform cortex, inferior frontal junction, superior frontal language area, ventral laryngeal–orofacial motor cortex and dorsal hand motor cortex. Regions were defined in each participant’s individualized Schaefer–Kong 900-parcel parcellation, and DCM time series were extracted from task-responsive voxels within each region of interest (ROI). Detailed ROI definitions and volumes of interest (VOIs) extraction procedures are provided in Supplementary Methods ROI definition for dynamic causal modelling analyses.

For each participant and task, we estimated one eight-node DCM. The endogenous connectivity matrix included self-connections for all nodes and bidirectional connections between area 55b and each of the seven other nodes; direct connections among non-55b nodes were not modelled. The lexical-processing contrast between each word task and its matched control condition was specified as a modulatory input on the bidirectional area 55b-centred connections. Driving inputs were assigned to sensory input regions according to task modality. DCMs were inverted and entered into a parametric empirical Bayes framework to estimate group-level effects and empirical-prior-updated subject-level modulatory parameters ^40,109^.

Parameters of interest were posterior expectations of lexical modulatory effects on each directed 55b-centred edge. For each participant, parameters were averaged across production and comprehension tasks and contrasted as (naming + writing)/2 − (listening + reading)/2. Edge-wise tests identified connections whose model-estimated lexical modulation differed between task domains; false discovery rate correction was applied across the tested directed 55b-centred edges.

#### Regional embedding within established cortical hierarchies

We asked whether area 55b occupied a local transition within broader cortical sensory-to-association hierarchies. For area 55b and comparison regions, we sampled three adjacent parcels spanning a sensorimotor- or sensory-adjacent parcel, the central parcel containing the target region, and an association-adjacent parcel. Ordered changes were quantified across six cortical hierarchy maps. This analysis establishes regional embedding around each target parcel; it does not directly test alignment between the hierarchy maps and the internal vertex-wise connectivity gradient within 55b.

#### Parcel sampling along the local sensory-to-association axis

Regions of interest were defined using the Schaefer–Kong 900-parcel parcellation. For area 55b and each comparison region, we identified three adjacent parcels arranged along a putative local sensory-to-association axis: a sensory- or sensorimotor-adjacent parcel, a central parcel corresponding to the target region, and an association-adjacent parcel. Parcel selections and anatomical criteria are detailed in the Supplementary Methods.

To assess whether any observed local hierarchical transitions were specific to conjunction-defined target regions rather than a generic property of the language network, we also defined three control regions within a canonical language network: inferior frontal gyrus (triangular part), mid–middle temporal gyrus and posterior middle temporal gyrus. For each control region, the same sampling strategy was applied, selecting a sensory-adjacent parcel, a central parcel and an association-adjacent parcel along the local cortical axis.

#### Cortical hierarchy maps

For each ROI, we examined how values from the sensory-adjacent, central and association-adjacent parcels changed across six cortical hierarchy maps spanning evolutionary, structural, transcriptomic, metabolic, cognitive, and composite dimensions of the sensory-to-association continuum. These maps were ordered from biological constraints to integrative functional organization. First, the evolutionary hierarchy was indexed by macaque-to-human cortical expansion, which quantifies the degree of cortical surface expansion from macaque to human, with lower values in primary sensory and motor cortices and higher values in transmodal association cortex ^41,110^. Second, the structural/myeloarchitectonic hierarchy was indexed by the T1w/T2w ratio, an in vivo proxy for intracortical myelin content, with heavily myelinated primary cortices at the sensory end of the hierarchy and lightly myelinated heteromodal regions at the association end ^111^. Third, the transcriptomic hierarchy was indexed by the first principal component of cortical gene-expression profiles derived from the Allen Human Brain Atlas, capturing a dominant gradient from sensory-biased to association-biased transcriptional signatures across cortex ^112^. Fourth, the metabolic hierarchy was indexed by PET-derived aerobic glycolysis, reflecting regional variation in non-oxidative glucose metabolism, which is typically lower in primary sensorimotor cortex and higher in association cortex ^113^. Fifth, the meta-analytic cognitive hierarchy was indexed by the first principal component of NeuroSynth term–activation decodings, summarizing large-scale functional specialization from lower-order sensorimotor functions to higher-order cognitive functions ^114^. Finally, the composite sensory-to-association hierarchy integrated structural, functional, metabolic and molecular features into a single cortex-wide ordering from primary sensory and motor regions to transmodal association cortex ^41^. Together, these six maps provided complementary biological and functional indices of where each parcel lay along the broader sensory-to-association hierarchy.

#### Quantification of monotonic hierarchical transitions

For each hierarchy map, vertexwise values were sampled within the sensory-adjacent, central, and association-adjacent parcels. Monotonic ordered differences were quantified using the Jonckheere–Terpstra test, with the expected order specified as sensory-adjacent < central < association-adjacent or the reverse, depending on map direction. The resulting Z statistic indexed local transition strength, and *P* < 10^−4^ was used as a stringent descriptive threshold. Because cortical vertices are spatially dependent, these tests were interpreted as map-level descriptions of ordered regional transitions rather than participant-level inferential tests.

#### Multidimensional comparison of local hierarchical transition strength

To compare the overall strength of local hierarchical transitions across ROIs, we summarized the six hierarchy-specific indices of local transition strength into a single multidimensional index and then compared this index between candidate and comparison regions using exact paired permutation tests. For each ROI, the absolute Jonckheere–Terpstra Z-statistics obtained from the six hierarchy maps were treated as the radii of a six-axis radar polygon, and polygon area was computed as

$$A=\frac{1}{2}\sin\left(\frac{2\pi }{n}\right)\sum_{i=1}^{n}r_{i}r_{i+1},$$

where $r_{i}=\left|\;Z_{i}\;\right|$, $r_{n+1}=r_{1}$, n=6. Larger values indicate stronger and more consistent local transitions across the six hierarchy dimensions.

For each pairwise comparison between a candidate region and a comparison ROI, an exact paired permutation test was performed across the six matched hierarchy maps. Under the null hypothesis of no difference in overall local hierarchical transition strength, the ∣Z∣ values for each hierarchy map were either retained or swapped between the two ROIs independently, yielding all 2^6^ = 64 possible reassignment patterns. For each reassignment, polygon areas were recomputed and the area difference $\Delta A=A_{\text{candidate}}-A_{\text{comparison}}$ was calculated to form the exact null distribution. One-tailed values were defined as the proportion of reassignment patterns for which $\Delta A_{\text{permutation}}\geq \Delta A_{\text{obsolute}}$. Statistical significance was set at *P* < 0.05.

#### Statistical analysis

Unless otherwise stated, group-level statistical analyses were performed using two-sided one-sample or paired-sample t-tests, according to the corresponding hypothesis. For paired-sample tests, outliers were identified separately for each planned contrast using a predefined criterion based on the paired-difference values. Participants whose paired-difference values fell outside the group mean ± 2.5 s.d. were excluded from that specific contrast before statistical inference. Because this criterion was applied independently to each contrast, the number of retained participants could vary across tests; the retained sample size for each paired test can be inferred from the reported degrees of freedom as N = df + 1. Multiple comparisons were controlled using FDR correction for families of related tests. Effect sizes were reported as Cohen’s *d*.

## Supplementary Material

Supplementary Files

This is a list of supplementary files associated with this preprint. Click to download.

• WuetalSupplementaryNNresubmissionfinal.docx

## Figures and Tables

**Table 1.** Modality-invariant lexical regions identified across comprehension and production tasks

Manuscript region	Schaefer–Kong parcel label	Glasser atlas label	Vertices	Minimum T across contrasts

IFJ	L ContA PFCl 5/7	Area IFJ L	99	3.51
SFL	L Language PFCd 2	Superior Frontal Language Area L	53	3.01
Area 55b	L SomMotB 8 /DorsAttnB PrCv 4	Area 55b L	95	3.02
Anterior fusiform gyrus	L VisualA ExStr	Ventral Fusiform L	68	3.86
